# Mass recalibration for desorption electrospray ionization mass spectrometry imaging using endogenous reference ions

**DOI:** 10.1186/s12859-022-04671-5

**Published:** 2022-04-15

**Authors:** Paolo Inglese, Helen Xuexia Huang, Vincen Wu, Matthew R. Lewis, Zoltan Takats

**Affiliations:** 1 National Phenome Centre, Imperial College London, Hammersmith Campus, IRDB Building, London, W12 0NN UK; 2 Section of Bioanalytical Chemistry, Division of Systems Medicine, Department of Metabolism, Digestion and Reproduction, Imperial College London, South Kensington Campus, London, SW7 2AZ UK

## Abstract

**Background:**

Mass spectrometry imaging (MSI) data often consist of tens of thousands of mass spectra collected from a sample surface. During the time necessary to perform a single acquisition, it is likely that uncontrollable factors alter the validity of the initial mass calibration of the instrument, resulting in mass errors of magnitude significantly larger than their theoretical values. This phenomenon has a two-fold detrimental effect: (a) it reduces the ability to interpret the results based on the observed signals, (b) it can affect the quality of the observed signal spatial distributions.

**Results:**

We present a post-acquisition computational method capable of reducing the observed mass drift by up to 60 ppm in biological samples, exploiting the presence of typical molecules with a known mass-to-charge ratio. The procedure, tested on time-of-flight and Orbitrap mass spectrometry analyzers interfaced to a desorption electrospray ionization (DESI) source, improves the molecular annotation quality and the spatial distributions of the detected ions.

**Conclusion:**

The presented method represents a robust and accurate tool for performing post-acquisition mass recalibration of DESI-MSI datasets and can help to increase the reliability of the molecular assignment and the data quality.

**Supplementary Information:**

The online version contains supplementary material available at 10.1186/s12859-022-04671-5.

## Introduction

In the last 20 years, mass spectrometry imaging (MSI) has attracted increasing attention as a technology capable of capturing molecular spatial patterns from sample surfaces. Among the available ionization sources, such as matrix-assisted laser desorption [1] (MALDI) or secondary ion mass spectrometry [2] (SIMS), desorption electrospray ionization [3] (DESI) has gained popularity and been widely applied thanks to its relatively simpler preparation process. A map of the detected ions’ spatial distribution is usually represented as an image and analyzed for identifying informative spatial patterns. MSI has been successfully deployed in various application areas, from cancer research [4–7] to pharmacology [8–12]. However, as an emerging technology, MSI still faces implementation challenges for controlling the quality of data produced [13–15]. The accuracy of mass-to-charge ratio (*m/z)* measurements under sustained use are chief among these concerns. MSI acquisitions often consist of tens of thousands of spectra (pixels), corresponding to several hours of operation, during which mass measurements can be subject to substantial drift. For example, a small image of 10 mm × 10 mm, acquired at a running speed of 1 scan/s, with a spatial resolution of 100 μm, requires about 2.8 h. Shifting mass measurements are detrimental to the analyzed spatial patterns and can result in erroneous chemical annotations. Additionally, as MSI relies on only a single dimension of separation for detected ions, errors of a few parts per million (ppm) can result in hundreds of candidate molecular identities, interpreting in terms of biochemical hypotheses a practical challenge.

For these reasons, it is crucial to develop quality control approaches for MSI that mitigate this issue and yield accurate *m/z* measurements with high precision during extended instrument use. Solutions successfully applied in more routine hyphenated techniques (e.g., liquid chromatography-mass spectrometry LC–MS) include periodic recalibration or more frequent calibration based on the measurement of one or more standard reference materials during the acquisition. Such an approach is infeasible for the current MSI technology since the acquisition is performed continuously while the probe moves across the sample surface. However, this approach may be emulated by adding the standard material to the sample [16]. Although this approach can be used to facilitate post-acquisition *m/z* correction of mass accuracy, concerns may arise about the unknown effect on the ionization efficiency caused by these exogenous molecules’ presence. In the specific case of DESI-MSI, usually, only one reference molecule (lock mass) is added to the solvent, which can be insufficient to capture the non-linear drift across the measured *m/z* range.

Another class of approaches relies on using endogenous or background peaks as candidate reference ions to estimate the mass shift occurring in each spectrum and map the measured *m/z* onto their corresponding recalibrated values [17]. This class of methods requires prior knowledge about the molecular content of the sample.

Boskamp et al. showed that endogenous signal (chemical noise) could improve the mass accuracy in MALDI-MSI of peptide datasets [18]. Recently, La Rocca et al. have presented an algorithm to recalibrate MSI datasets using a linear fit on the observed mass errors from endogenous biological peaks [19]. In their method, they treated each spectrum as independent.

Here, we present a different approach based on a fixed set of reference ions across the entire MSI dataset, in which the individual mass shifts are modeled as a time series. The advantages of global references are that they (1) define a fixed boundary for the *m/z* extrapolation, (2) improve evaluation of the quality of the matching procedure by visualizing their spatial distribution, and (3) increase the robustness of the matching process as a time-dependent model.

The approach leverages the ubiquity of common structural components of tissues as dependable sources of reference values, using them as multiple points to estimate error and correct all observed masses in each spectrum. The approach is demonstrated on biological tissue datasets acquired using a DESI-MSI source interfaced with time-of-flight (TOF) or Orbitrap ion analyzers, in both positive (ES+) and negative (ES−) ion mode.

For simplicity, we will refer to *m/z* values as “masses” throughout the manuscript.

## DESI-MSI datasets

### In-house DESI-MSI datasets

DESI-MSI data of six tissue sections from mouse (mus musculus) brain and pig (sus domesticus) liver were acquired using a Waters Xevo G2-XS QToF mass spectrometer.

Three brains of a C57BL/6 mouse model were purchased from Charles River Laboratories, while pig liver samples were obtained from a local supermarket.

We used the sample preparation procedure and DESI parameters reported in Tillner et al. [20]. The mouse brain samples were scanned at a rate of 75 μm/s horizontally, while the pig liver sample was scanned at a rate of 100 μm/s.

The RAW spectra from the six acquired TOF DESI-MSI were first converted into imzML using the MassLynx SDK (v4.7.0) and Python’s pyimzML package.^1^ Then, they were filtered from the baseline noise using a modified version of the *kneedle* algorithm [21] (Additional file 1: Section S1) and smoothed using a Savitzky-Golay kernel convolution. Centroided peaks were detected and used as input for the recalibration procedure (Additional file 1: Section S2). Throughout the manuscript, we will refer to spectrum and list of centroid peaks as synonyms.

### Public DESI-MSI datasets

Twenty-four DESI-MSI datasets were downloaded from the public service METASPACE.^2^ We considered various tissue types analyzed with either Orbitrap or TOF ion analyzers. Details about the datasets can be found in Additional file 1: Table S1.

The datasets consisted of centroided RAW peaks. No peak detection or denoising was applied.

## Methods

### Selection of sample pixels

The first step of the presented workflow consists of determining the pixels associated with the biological sample. Since our method uses endogenous molecules that are expected to be detected by DESI-MS in tissue, it is crucial to remove all pixels that do not correspond to tissue-related signals.

The procedure aims at discriminating the sample-related from off-sample pixels, using a supervised classifier trained on a set of user-defined labeled pixels.

We first define the set of features from the RAW peaks, applying a uniform binning with a bin size of 1 m*/z*. Subsequently, through a graphical user interface (GUI), the user manually labels a set of pixels as either ‘sample’ or ‘background’ (Additional file 1: Fig. S2). Then, a linear support vector machines (SVM) model is fitted on this set of features and labels and used to predict all pixels’ labels, providing a binary map of the region-of-interest (ROI). The user can manually refine the segmentation through the GUI. Finally, connected regions (8-neighborhood) smaller than a given size are assigned to the background class. In all the experiments, we set the size threshold equal to 50 pixels.

The ROI mask is saved in a comma-separated values (CSV) file to be used in the workflow’s following steps.

### General recalibration workflow

In this section, a general description of the recalibration method is given, with details reported in the following sections.

Let us consider a DESI-MSI dataset representing a collection of *N* sequentially acquired spectra (where *N* = *N*^(ROI)^ + *N*^(off)^*)* is the sum of the number of sample ROI and off-sample pixels).

Given an observed mass spectrum, the calibration procedure aims to estimate the theoretical (“calibrated”) masses from the observed masses of the detected ions.

One common strategy is based on reference points, called *lock masses* [22]. Given a mass spectrum from a pixel $p$, this method assumes a statistical model *g* between the theoretical ${\mathbf{M}}_{p}=({M}_{k,p})$ and observed ${\mathbf{M}}_{p}^{\#}=\left({M}_{k,p}^{\#}\right)$ masses:

1 $${\varvec{M}}_{p} = g_{p} \left( {{\mathbf{M}}_{p}^{\# } ,{{\varvec{\uptheta}}}_{p} } \right)$$

where the index $p$ indicates that the observed and theoretical masses, the model *g* and its parameters ${\varvec{\uptheta}}$ are spectrum (or pixel)-specific.

Given a set of reference masses and their observed values, the parameters are fitted from data, and the model ${g}_{p}$ is used to predict the calibrated values of all detected masses.

When using endogenous signals as a reference, lock masses can be spectrum-specific [19] ($\mathbf{M}={\mathbf{M}}_{p}$) or be defined globally. Our method uses the latter approach.

When using a global set of reference masses, the models ${g}_{p}$ can only be fitted if the reference masses are observed in all pixels $p$. However, due to the molecular heterogeneity of biological samples, this property is not guaranteed. To overcome this challenge, for all reference masses $\mathbf{M}$, we model their observed values in the pixels $p$ as a smooth function of the acquisition time (or, equivalently, the pixel order). This is equivalent to assuming that the observed masses ${\mathbf{M}}^{\#}$ depend on the actual conditions of the instrument, and that these smoothly vary in a controlled environment. In practice, we model each mass in ${\mathbf{M}}^{\#}$ as a time series, where the pixel order is a proxy variable for the acquisition time.

The temporal trends of the masses’ shifts are fitted as follows. Given a the theoretical value of a reference mass ${M}_{k}\in \mathbf{M}$, let ${\mathbf{P}}_{M}=\left({p}_{1},{p}_{2},\dots \right)=({p}_{i})$ be the subset of ROI pixels where it is observed with mass ${\mathbf{M}}_{k}^{\#}=\left({M}_{k,{p}_{1}}^{\#},{M}_{k,{p}_{2}}^{\#},\dots \right)=\left({M}_{k,{p}_{i}}^{\#}\right)$. We model the time series trend using a generalized additive model (GAM) [23]:

2 $$\begin{aligned} & M_{{k,p_{i} }}^{\# } |\eta_{{k,p_{i} }} ,\sigma^{2} \sim \,{\text{Normal}}\left( {\eta_{{k,p_{i} }} ,\sigma_{k}^{2} } \right) \\ & \eta_{{k,p_{i} }} = \beta_{0,k} + \mathop \sum \limits_{j = 1}^{J} s_{j,k} \left( {p_{i} } \right) \\ \end{aligned}$$

where $s_{j,k}$ are penalized cubic spline functions and *J* is equal to 20.

The procedure, repeated for all ${M}_{k}\in \mathbf{M}$, separately, provides the trend models for all reference masses. As for any regression models, if ${\mathbf{P}}_{M}$ corresponds to ‘a large portion’ of the ROI, the fitted model can accurately predict the values of each database mass, ${\widehat{\mathbf{M}}}_{k}={{\varvec{\upeta}}}_{k}=\left({\eta }_{k,{p}_{i}}\right)$,^3^ that would have been observed (up to a random error term) in all ROI pixels ${p}_{i}$, given the instrumental condition at the acquisition time.

In the following section, we describe the procedure to detect and select the reference masses for the shift trends estimation.

### Selection of reference masses and shift trend modelling

Since the molecular composition of the sample is largely unknown, it is practically impossible to define a general set of lock masses that works for all datasets. Before describing the procedure aimed at identifying them, we need to introduce properties that define ‘good’ endogenous reference masses.

The candidate reference mass is optimal if: (a) it is detected in a ‘large’ portion of the sample, (b) its peak is ‘isolated’ in the *m/z* space. Property (a) allows us to use the model described in Eq. 2, while property (b) reduces the uncertainty of the matching procedure.

The procedure aimed at establishing if an observed mass matches a database mass is described in the following. As the reference ions consist of molecules that are expected to be found in tissue samples and be detected by DESI-MS, we perform a database search to determine the list of candidates. The database ${\varvec{\Omega}}$ includes phospholipids, fatty acids, mono/di/triglycerides, cholesterols, and ceramides from Lipidmaps [24] and Human Metabolite DataBase [25] (HMDB) databases. Deprotonated and chlorine adducts ([M − H]^−^, [M + Cl]^−^) are considered for negative polarity mode, while protonated, sodium and potassium adducts ([M + H]^+^, [M + Na]^+^, [M + K]^+^) are considered for positive polarity mode.

Given a database mass $M\in{\varvec{\Omega}}$, a mass ${m}_{i}$ among observed masses in pixel ${p}_{i}$ is considered a candidate match if ${m}_{i}\in \left[M-\lambda ,M+\lambda \right]$, where $\lambda =W\times M\times {10}^{-6}$ is the mass distance from $M$ corresponding to the relative error $W$ in ppm units. All masses satisfying the condition are considered possible matches, meaning that more matches per pixel can be found. For each candidate match, we retrieve its peak index ${\pi }_{i}$, observed mass ${M}_{i}^{\#}={m}_{i}$, and intensity ${\iota }_{i}^{\#}$.

To satisfy property (a), for all $M\in{\varvec{\Omega}}$, we remove the candidate matched peaks that are detected in less than 75% of the sample ROI pixels.

Subsequently, we further filter the list of candidate reference ions using a method based on a kernel density estimator (KDE) following a similar procedure to that described in Smirnov et al. [26] If candidate reference peaks for *M* are represented as points with coordinates $\mathbf{x}=\left(x,y\right)=\left({p}_{i}, {M}_{i}^{\#}\right)$, we assume that highly dense connected regions represent the shift trends of *M*.

Given a candidate reference mass $M\in {\varvec{\upmu}}\subseteq{\varvec{\Omega}}$ and its observed values ${\mathbf{M}}^{\#}=\left({M}_{i}^{\#}\right)$, we estimate the density ${f}_{h}=\frac{{\sum }_{i}K\left(\frac{x-{x}_{i}}{h}\right)}{nh}$ of the $\left(x,y\right)$-points using a 2D Fast Fourier Transform (FFT) KDE [27], with a triangular kernel *K*, defined as

3 $$K\left(\mathbf{x}\right)=1-\left|\mathbf{x}\right|, \left|\mathbf{x}\right|\le 1$$

The kernel is fitted on the coordinates of the points scaled to $\left[\mathrm{0,1}\right]$ interval.

The triangular kernel is chosen because of its computational efficiency. The kernel bandwidth is set to $h=2.576\times \sigma \times {N}^{-1/5}$, where *σ* represents the standard deviation of the whole set of points and *N* represents the number of points $\mathbf{x}$ [28]. The kernel is fitted on a regular grid of size $G\times G$, with $G=1024$.

Given the estimated 2D density ${\widehat{f}}_{h}={\widehat{f}}_{h}\left(x,y\right)$, we identify the curve passing through its local maxima as follows. For each $x^{\prime } \in \left\{ {1, \ldots ,G} \right\}$, we determine the local density maximum $y^{\prime } = \mathop {\arg \max }\limits_{{x = x^{\prime } }} \hat{f}_{h} \left( {x,y} \right)$. If $\hat{f}_{h} \left( {x^{\prime } ,y} \right) = 0$ for all $y$, no maximum is considered for that value of $x^{\prime }$. Subsequently, a cubic spline *S*(*x*) is fitted on the vector of local maxima $\left(\widehat{x},\widehat{y}\right)$ after transforming them back to the original (pixel, mass)-space. This step allows to determine a smooth model along the detected local KDE maxima. Using the predicted spline values $S\left({p}_{i}\right)$ for all ${p}_{i}$, we calculate the absolute residuals ${r}_{i}$ and the dispersion ${d}_{i}$:

4 $$\begin{array}{*{20}c} {r_{i} = \left| {M_{i}^{\# } - S\left( {p_{i} } \right)} \right|} \\ \end{array}$$

5 $$\begin{array}{*{20}c} {d_{i} = \frac{{2 \times {\text{mad}}\left( {\mathbf{r}} \right)}}{{S\left( {p_{i} } \right)}} \times 10^{6} } \\ \end{array}$$

**Fig. 1:**
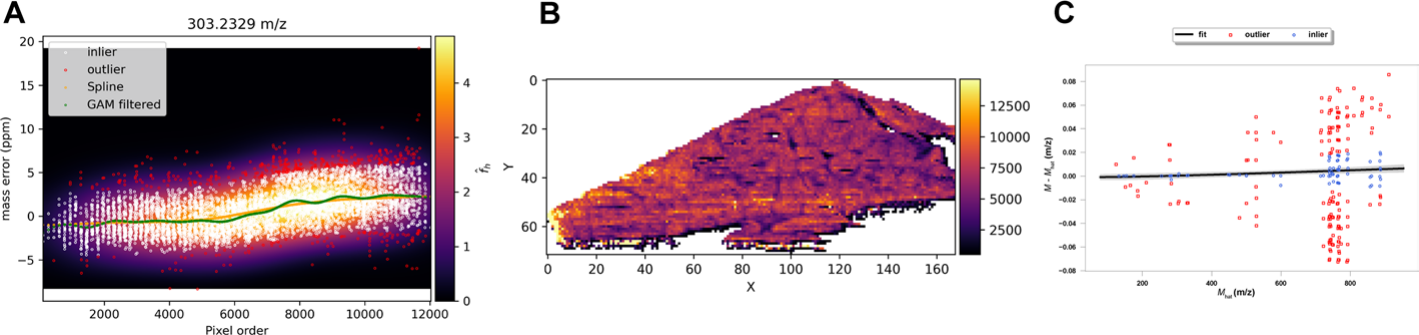
Example of the recalibration process for the Orbitrap dataset liver ES-. **A** FFT KDE is fitted from the matched masses for a candidate reference (303.2329 m/z). The points represent the difference in m/z between the observed and the theoretical values of the reference. The orange curve passing represents the spline fitted on the density local maxima. The GAM predictions are plotted in green. Points in white represent the inliers used to fit the GAM, while outliers are plotted in red. **B** The intensity corresponding to the matched points can be plotted to reveal their spatial distribution. This allows validating the consistency of the selected matches visually. **C** Finally, a regression model is fitted in each pixel using the only reference masses with similar errors in ppm (blue circles) within the residual intervals defined by the filter (Eqs. 6–7). In the case of TOF data, the degree of the polynomial corresponds to the smallest BIC value. The predicted values (black line, the grey bands represent 95% confidence intervals) are used to correct all observed masses in the pixel. In **C**, for clarity, the mass errors are plotted on the y-axis instead of the predicted observed masses

Points with ${r}_{i}\ge 2\times \mathrm{mad}(\mathbf{r})$, $\mathbf{r}=\left({r}_{i}\right),$ where “*mad*” is the median absolute deviation multiplied by the inverse of cumulative Normal distribution $1/{\Phi }^{-1}(3/4)\approx 1.4826$, are considered outliers and removed from the list of matched peaks (Fig. 1A).

Finally, the candidate reference $M$ is kept if: the number of distinct inlier pixels is greater or equal than 75% of the ROI size and $\underset{i}{\mathrm{max}}({d}_{i})\le 10$ ppm. In this way, we select isolated signals (property (b)). The choice of a threshold equal to 10 ppm for the dispersion around the fitted spline is based on the expected scattering of the mass shifts due to intrinsic measurement noise, and it can be customized by the user.

The procedure is repeated for all candidate reference masses.

The final set of reference masses is denoted as ${\mathbf{M}}^{*}$. The mass shift GAM trends (Eq. 2) for each ${M}^{*}\in {\mathbf{M}}^{*}$ are fitted using the corresponding inlier points, and the models are used to predict the observed values $\widehat{\mathbf{M}}=\left({\widehat{M}}_{i}\right)$ in all ROI pixels (Fig. 1A). The use of GAM with penalized cubic splines aims at capturing the details of the shift trends better than the single spline regression. The intensities ${\iota }_{i}^{\#}$ of the matched peaks can be plotted to reveal their spatial distribution (Fig. 1B). Visual inspection provides additional validation of the consistency of the matched peaks. When multiple matches are available per pixel, the intensities of the peaks with the smallest ${r}_{i}$ are plotted.

### Pixel-wise mass recalibration

In this section, we will describe the procedure used to finally recalibrate the masses detected in all ROI pixels.

Although the selected reference ions have passed the filtering procedure, mismatched peaks may still be present if: (a) same peaks fall within two search windows so that they are assigned to two reference ions, (b) shifted peaks fall within the search window by chance (especially if the mass shift is large).

To reduce the chance of using mismatched reference ions masses, we apply a pixel-specific mass filter, based on the assumption that most of the true matches share a similar relative mass error. The details of the filtering procedure are reported in Additional file 1: Section S4.

Once the set of reference masses${\mathbf{M}}_{p}^{\mathbf{*}}=\left({M}_{1,p}^{*},{M}_{2,p}^{*}\dots \right)=\left({M}_{k,p}^{*}\right)$^4^ is determined for pixel *p* together with the values predicted by GAMs, we fit a model *g* (Eq. 1) specific for each type of ion analyzer (Additional file 1: Section S3). For Orbitrap analyzers, we fit a linear regression model [29]

6 $$\begin{aligned} & M_{k,p}^{*} \sim {\text{Normal}}\left( {\eta_{k,p} ,\sigma_{p}^{2} } \right) \\ & \eta_{k,p} = \beta_{0,p} + \beta_{1,p} \hat{M}_{k,p}^{\# } \\ \end{aligned}$$

Instead, for the TOF analyzer, we use a polynomial regression model [30, 31]

7 $$\begin{aligned} & \sqrt {M_{k,p}^{*} } \sim {\text{Normal}}\left( {\eta_{k,p} ,\sigma_{p}^{2} } \right) \\ & \eta_{k,p} = \beta_{0,p} + \mathop \sum \limits_{d = 1}^{D} \beta_{d,p} \sqrt {\left( {\hat{M}_{k,p}^{\# } } \right)^{d} } \\ \end{aligned}$$

where the optimal polynomial degree *D*, *D* ≤ 5, corresponds to the smallest Bayesian Information Criterion (BIC) [32].

The fitted model is then used to predict the calibrated mass for all the detected ions in the specific pixel (Fig. 1C).

Schematically, the mass recalibration method follows these steps:

1. ROI pixels and reference mass database are given.
2. Search reference masses within a user-defined tolerance (in ppm units).
3. Keep masses with hits in at least 75% of ROI pixels.
4. For each kept mass, fit a 2D FFT KDE of the pixels and observed mass values:
  Fit a spline regression on the KDE local maxima.
  Identify the inliers and outliers from the spline residuals.
  Exclude the matched mass if the inliers points are less than 75% of ROI pixels or their residuals have a too large “mad” (Eq. 5).
  If the reference is kept, fit a GAM using the inlier points.
  Predict the observed masses in all ROI pixels using the fitted GAM.
5. For all ROI pixels:
  Select the masses predicted by GAM in the pixel that have a common relative mass error.
  Fit a linear or polynomial regression using the selected predicted masses and the corresponding theoretical values.
  Use the regression model to predict the recalibrated masses in the pixel.

### Recalibration method from La Rocca et al.

We compared the performance of the presented method with that described in La Rocca et al. (La Rocca) [19].

The ROI pixels masses of the DESI-MSI datasets were recalibrated using the default parameters, as described in their original work: bandwidth for the density estimation function (“st”) equal to 0.0005, Da tolerance for the identifications (“tl”) equal to 0.01, limit in Da for hits selection (“lm”) equal to 0.002. To perform a fair comparison with our method, we used the same database described in "Selection of reference masses and shift trend modelling" section

## Results and discussion

As a first experiment, we tested the robustness of the pixel-wise recalibration model and the reference mass filtering described in Additional file 1: Section S4. We simulated a series of pixel-wise reference masses and their observed values following the method described in Additional file 1: Section S5 (Additional file 1: Fig. S1). In all simulations, we observed a significant reduction of the median relative error, in both Orbitrap and TOF models. Also, the simulations confirmed the reduction of the heteroskedasticity of the residuals (Additional file 1: Fig. S3).

We then tested the DESI-MSI datasets after removing the pixels outside of the ROI as described in "Selection of sample pixels".

All recalibration parameters were kept fixed within the Orbitrap or TOF datasets, as described in the "Methods" section. For Orbitrap datasets, we used a search window $W=20$ ppm, while for TOF datasets we used $W=100$ ppm.

FFT KDEs for matched points were fitted using the “KDEpy” package for Python^5^ (v. 1.1.0). The cubic splines are fitted using the function *UnivariateSpline* available in “SciPy” package for Python (v 1.7.1) [33]. The smoothing parameter *s* of the function was determined by fivefold cross-validation, among 30 values from 0.00001 to 0.1 evenly spaced in the log_10_ scale, corresponding to the smallest mean squared error.

**Fig. 2:**
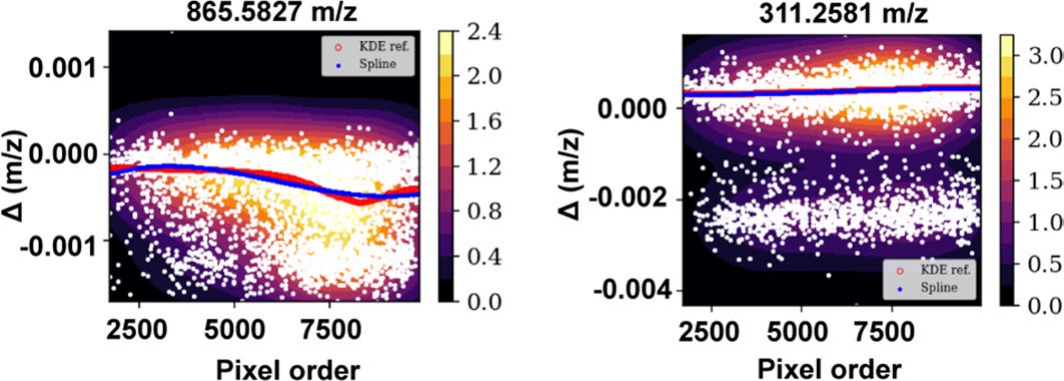
Left: Two peaks are too close to be resolved by the KDE. This reference is correctly discarded because of the large dispersion around the regression spline. Right: in this case, the KDE can resolve the two close peaks. Here, the method correctly fits the spline on the points belonging to one of the peaks. After filtering the outliers, the dispersion is below the threshold

The KDE-based model of the reference mass shift showed its robustness in the case of close peaks, correctly discarding wrongly matched peaks (Fig. 2).

The GAMs were fitted using the pyGAM package (v. 0.8.0) for Python [34]. The spline penalization parameter was chosen among eleven values varying between 0.001 and 1000 evenly spaced in the log_10_ scale, corresponding to the smallest generalized cross-validation value (GCV) [35].

In all tested DESI-MSI datasets, except for Orbitrap pancreas ES-, the selected reference masses for the pixel-wise recalibration well covered their acquisition *m/z* range (Additional file 1: Figs. S4–S5). The number of reference masses used for the recalibration varied between 15 and 174 (Additional file 1: Tables S2–S3).

In general, the reference masses were more concentrated in the *m/z* ranges below 400 and above 600, corresponding to small molecules and phospholipids, respectively (annotations are available online, see “Code and data availability” section).

In nine TOF datasets, there were pixels in which the optimal recalibration model was polynomial with a degree greater or equal to two. In particular, the distribution of the regression models coefficients revealed that the TOF mass shifts were greater than Orbitrap (Orbitrap error within 5 ppm, while TOF error up to 65 ppm) (Additional file 1: Table S4–S5). This observation may indicate a higher tendency of one class of analyzers to be subject to the changes of the environmental conditions, although these may depend on the unknown actual instrumental conditions for each dataset.

To evaluate the recalibration efficacy, we looked at two main effects: (1) the number of putative molecular annotations, (2) mass error of test ions.

The number of putative annotations was generated using the METASPACE website. METASPACE assigns molecule identities to MSI datasets based on a metabolite-signal match score (MSM) calculated from spectral and spatial measures and an FDR-estimation using a decoy strategy. Each assignment is characterized by an FDR equal to 5%, 10%, 20%, and 50%. The annotation is performed by database search. Because of the biological nature of the analyzed samples, we selected the following four databases among those available: (a) “ChEBI 2018-01”, (b) “CoreMetabolome v3”, (c) “HMDB v4”, (d) “LipidMaps 2017-12-12”.

We only considered the number of unique annotated *m/z* values corresponding to the stringent criterion of FDR = 5% (column “mz” of the annotation tables). We considered the unique annotated *m/z* values corresponding to the four databases combined. The annotations were performed using the ROI filtered raw and recalibrated peaks without applying any peak binning or spatial filter.

We checked whether the largest number of unique annotations was found in the original or recalibrated dataset.

In twenty-two cases ($\approx$ 73%), the recalibrated dataset received a larger number of assignments compared to the raw dataset when considering the combined databases. The recalibration increased the number of annotations by up to 281. In contrast, the largest decrease was observed in Orbitrap necrosis ES+ with 68 fewer annotations, equivalent to about 12.5% of the original annotations (Table 1)**.** In this and pancreas ES+, where we observed the largest decrease of annotated molecules, the missing annotations received and FDR = 10% in the recalibrated datasets, due to a small number of missing pixels. Because no specific pattern was associated to these empty pixels, we hypothesize that they are due to statistical noise (Additional file 1: Fig. S6).

**Table 1:**
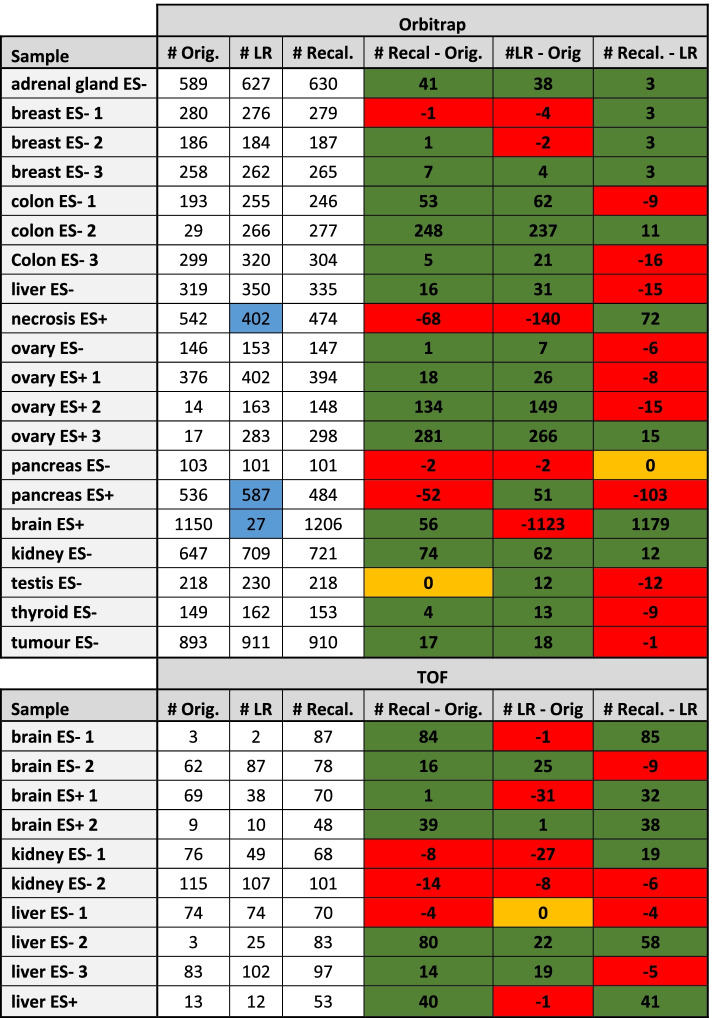
Number of annotated molecules by METASPACE using the combined set of databases (FDR = 5%)

The number of annotations for the original (non-recalibrated), processed with La Rocca (LR) and processed with our method datasets are reported in the first three columns respectively. The last three columns represent the difference between the number of annotations in the recalibrated datasets and the original dataset and between the two recalibration methods. Cases where our method results in a greater number of annotations are shaded in green, otherwise in red. In orange, the cases where the number of annotations is equal. The three datasets where we observed split annotations in LR are coloured in light blue

**Fig. 3:**
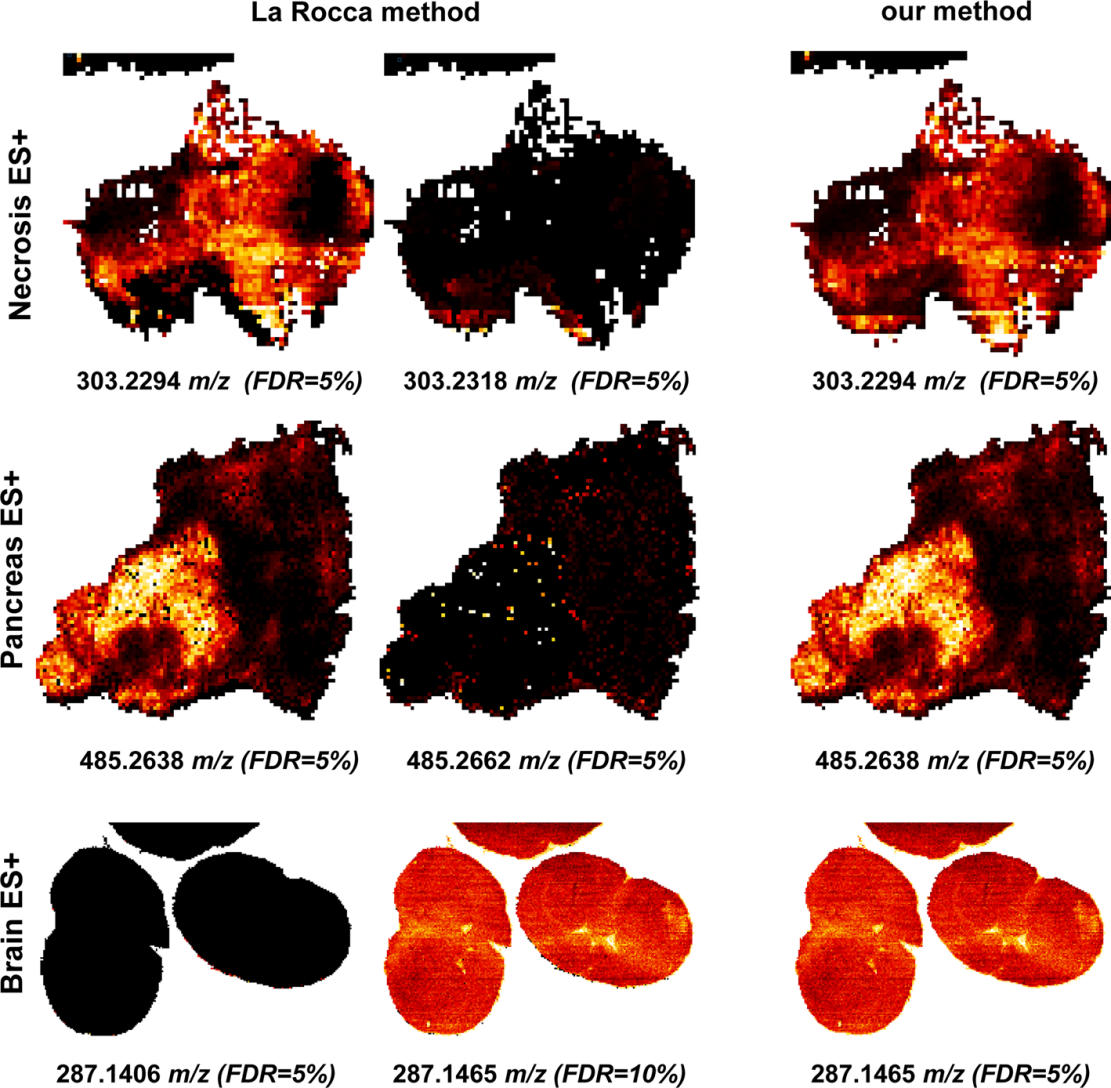
Examples of split annotations in La Rocca method, that instead are correctly annotated with our method. In some cases, the additional annotation was assigned with an FDR of 5%, in other cases, it received an FDR of 10%. This resulted in a possible inflation of the number of annotated molecules in the datasets processed using La Rocca

Using La Rocca method, in three Orbitrap datasets: “necrosis ES+”, “pancreas ES+” and “brain ES+”, we observed annotations corresponding to the same image split in two. These were instead correctly annotated as a single molecule when processed with our method (Fig. 3). Evidently, this issue affected the final number of annotated molecules in the datasets processed using La Rocca method. In the case of pancreas ES+, it resulted in an inflation of the number of annotated molecules, since both the split parts received an FDR = 0.05, while in the other two affected datasets, one of the split parts received an FDR = 0.1. This result confirmed that having a global model (time-dependent shift trend) increases the robustness of the final recalibration models, compared to considering each pixel as an independent sample.

In all other datasets, we observed a balanced outcome, with a slightly better performance of La Rocca in the Orbitrap datasets, and significantly better performance of our method in the TOF datasets. Specifically, in four TOF datasets, we observed that La Rocca was unable to improve the number of annotated molecules as our method (Table 1).

Subsequently, we tested the mass accuracy of a set of test masses to evaluate the effect of the recalibration. We followed the same idea described in Boskamp et al. [18]. Since the actual molecular content of a sample was unknown, we compared the mass accuracy of a list of ions expected to be detected by DESI-MSI in biological tissue samples.

The test ions list was generated from the available annotated molecules in the public datasets of METASPACE. The list consisted of the monoisotopic form plus the first three identified isotopes detected in more than 10% of the datasets from the same tissue type and ion mode of the DESI-MSI dataset. We used a Python script from LaRocca et al.^6^ (version available in June 2021) to generate the list of candidate test masses. The mass values common to the set used for fitting the pixel-wise recalibration models were excluded. These masses could either belong to different databases from those used for the mass recalibration or could have been skipped during the recalibration due to the more stringent filtering (limit of mass error dispersion).

In all datasets, the test masses covered the acquisition *m/z* range, confirming that they represented a good set of probes for evaluating the recalibration effects (Additional file 1: Figs. S3–S4).

**Fig. 4:**
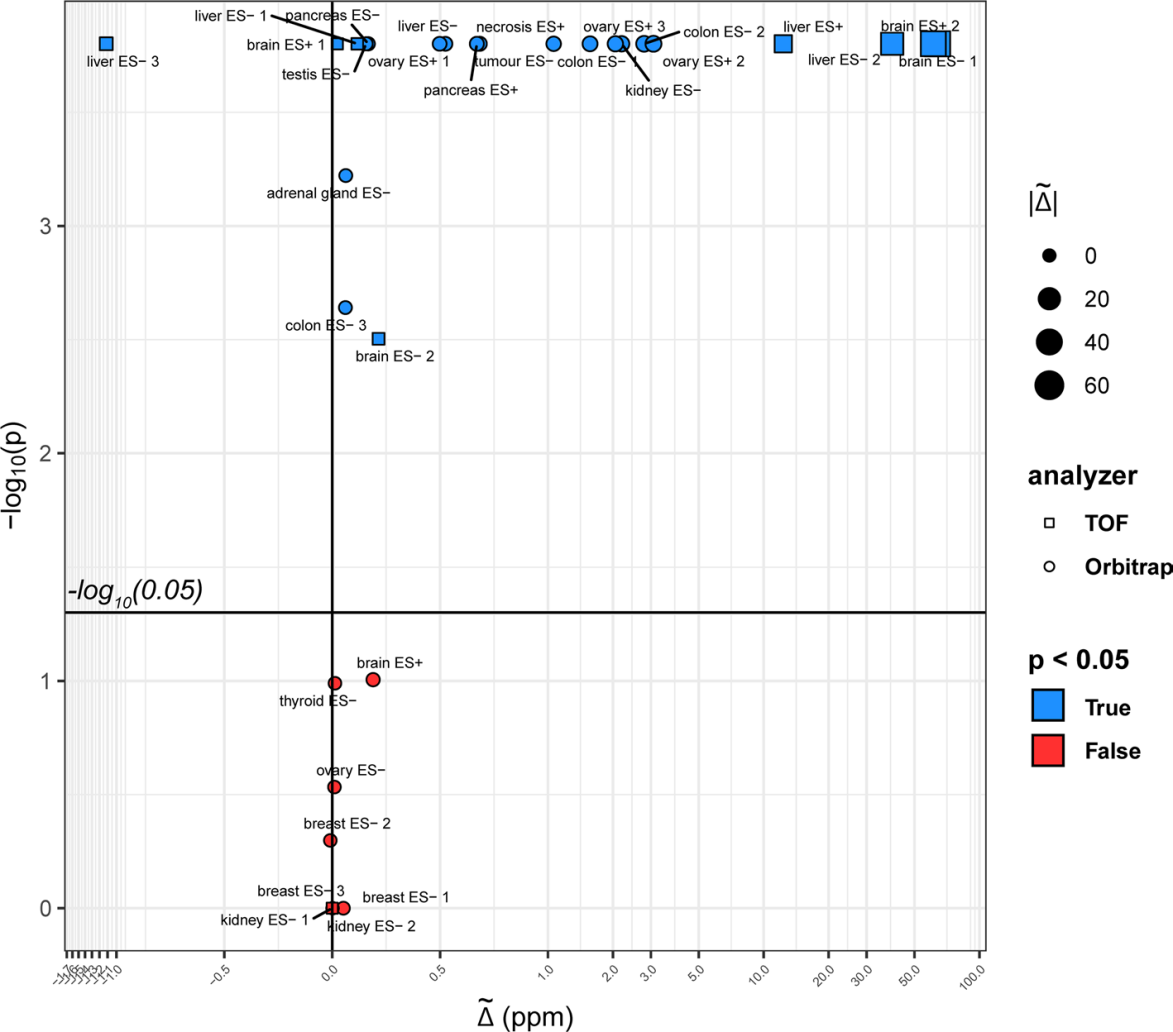
Scatter plot representing the bootstrapping results for the median difference between the mass relative errors in the original and recalibrated datasets. Each dataset is characterized by its $\stackrel{\sim }{\Delta }$ value (calculated following the method described in Additional file 1: Section S6) and the significance value of the bootstrapping test. Most datasets show a significant reduction of the test masses absolute error, with values up to about 60 ppm

Using the procedure described in Additional file 1: Section S6, we selected the test masses within +/− 2.5 ppm from the most abundant relative errors and calculated the median of the pixel-wise median difference between the absolute mass errors $\stackrel{\sim }{\Delta }$. We tested if we could reject the null hypothesis ${\mathrm{H}}_{0}: \stackrel{\sim }{\Delta }=0$, using bootstrapping (number of repetitions equal to 10,000). We observed in twenty datasets a significant (Benjamini–Hochberg corrected *p* value < 0.05) decrease of the median relative error after the recalibration, with values varying between 0.02 and 64 ppm. In all these datasets, the final median relative error was below 3.7 ppm for Orbitrap, and below 6 ppm for TOF. In one case (TOF liver ES− 3), the median relative error increased by about 1.8 ppm, with a final median relative error equal to 4 ppm. In nine datasets, $\stackrel{\sim }{\Delta }$ was not significantly different from zero (Fig. 4, Additional file 1: Tables S6–S7).

Consistently, the improvement of mass accuracy observed in the TOF datasets corresponded to a substantial increase of annotated molecules.

**Fig. 5:**
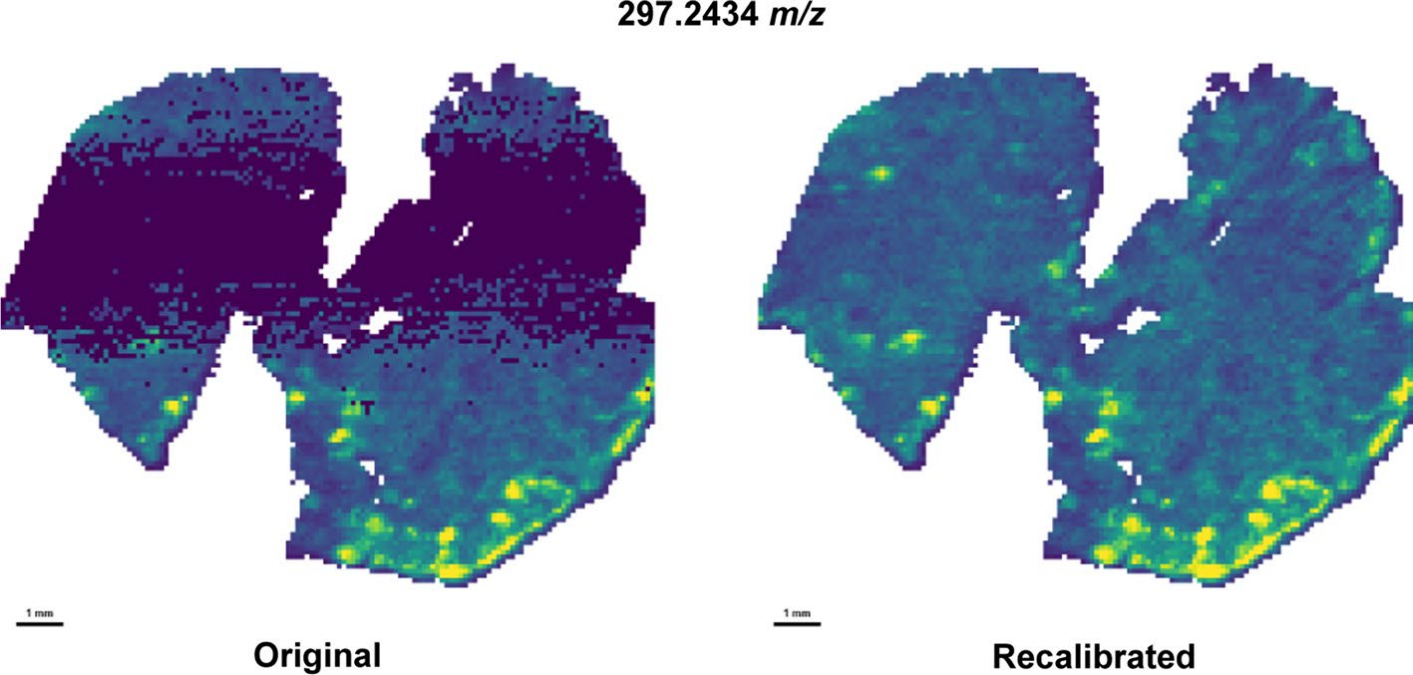
Example of the effect of recalibration on the quality of an assigned metabolites’ spatial distribution by METASPACE for the ORBITRAP colon ES− 2. The original dataset shows a band of missing intensities caused by the masses’ nonlinear distribution across the pixels, which is corrected in the recalibrated data

Furthermore, as already discussed before, the recalibration improved the ion image quality. After removing the mass shifts, all pixels correctly displayed the expected molecular spatial distributions (Fig. 5).

When compared with La Rocca method, our method performed slightly worse in the Orbitrap datasets, with a difference below 1 ppm in all cases (Additional file 1: Table S8). However, in the TOF datasets, our method outperformed La Rocca, which failed to improve the mass accuracies when the original relative errors were above 15 ppm (Additional file 1: Table S9). Since in these cases, the original error was large, we tested La Rocca with an optional set of parameters provided by the authors on GitHub for “low precision analyzers”: “st” = 0.03, “tl” = 0.8, “lm” = 0.08. Using these parameters, we observed an improvement in the datasets “brain ES− 1” and “liver ES− 2”, passing from 69 to 6 ppm and from 46 to 21 ppm, respectively. However, in all datasets, La Rocca still performed worse than our method, with also a reduction of the mass accuracy in some datasets (Additional file 1: Table S10).

We tested the effect of using polynomial models for the mass recalibration of the TOF datasets, forcing a linear model. The results of the relative mass errors for the test masses were close to those obtained in the original models (Additional file 1: Table S11) suggesting that also linear models would be feasible for TOF datasets.

We found that among the Orbitrap datasets, three masses were always used to fit the recalibration models: 253.2173 m*/z*, 269.2486 m*/z*, 279.2329 m*/z* for ES-, and 441.2975 m*/z*, 457.2715 m*/z*, 469.3288 m*/z* for ES+. Among the TOF datasets, we observed four masses always used as a reference for ES−: 255.2329 m*/z*, 281.2486 m*/z*, 283.2642 m*/z* and 303.2329 m*/z*. For ES+, we observed 14 masses always used: 309.2036 m*/z,* 621.4855 m*/z*, 621.4878 m*/z*, 649.5168 m*/z*, 649.5191 m*/z* 713.4518 m*/z,* 723.4935 m*/z,* 739.4675 m*/z,* 782.5670 m*/z*. 798.54077 m*/z,* 798.5410 m*/z,* 820.5253 m*/z,* 824.5566 m*/z,* 826.5721 m*/z*. Although these could be used as the only reference masses, using a database is a more robust solution for unknown MSI datasets.

## Conclusion

Despite its enormous potential to capture the spatial characteristics of the metabolic and proteomic mechanisms in a wide range of biological samples, MSI remains a relatively young technology. Advancements in fundamental data quality control are necessary to transform it into a more reliable approach.

Accuracy of measured masses is particularly challenging in imaging approaches since usually a run consists of tens of thousands of pixels and may require several hours to complete. Thus, the changes of the instrumental condition during this time may invalidate its initial configuration, resulting in mass drifts that correlate with time or pixel order.

Here, we have presented a computational workflow that exploits the presence of typical biological molecules in most of the sample spectra and uses them as a set of reference ions for applying a spectra-wise lock mass correction.

Using a global set of reference ions for the entire MSI dataset allowed us to test the assignment’s validity by visualizing the corresponding spatial distributions. Furthermore, we modeled the mass drifts as a smooth function of the acquisition time, as expected in acquisitions occurring in a controlled environment. Thus, these models represent an additional level of evidence about the assignments’ correctness.

FFT KDE-based reference match filtering, together with GAMs, proved robust against outliers and false-positive reference ions, for instance, in the presence of close peaks with a complementary spatial distribution to that of the actual reference ions peaks.

We showed that the presented approach could improve the mass accuracy, removing the nonlinear fluctuations of the measured masses. Additionally, our approach improved the molecular assignment to the detected peaks, using state-of-the-art molecular annotation methods, such as METASPACE, and their quality in terms of assigned spatial distributions.

In datasets with already accurate masses, the recalibration can introduce errors due to its statistical nature. For this reason, it is crucial to evaluate the test masses accuracies before and after performing the recalibration, using the methods described here.

We employed pixel-wise recalibration models specific to the physics of the MS analyzer. This is an essential aspect of the procedure since these characteristics influence the statistical properties of the signal generated from the detected ions.

It is also important to underline that we designed the mass shift models to be simple, considering only the time-dependency of the acquisition. Unfortunately, this means that several complex aspects of the phenomena driving the mass drifts are not captured. This is a necessary trade-off between generality and reduction of the risk of overfitting. More realistic models may be designed if additional variables are tracked, such as temperature, humidity, just to name a few.

We showed that computational models could effectively reduce the mass drifts that affect MSI datasets. However, it is crucial to stress that they can represent a temporary solution to this problem until technological solutions capable of performing an accurate online calibration will not be available.

When compared with a similar method, the results confirmed that considering the non-independence of the ion mass shifts increased the accuracy of the results, avoiding split annotations and being able to correct substantial relative mass errors.

The presented work has limitations. Although we used an extensive list of publicly available molecular masses as references, some peculiar molecules for unknown samples may be missing. For this reason, building a database of accurately identified molecules in previous studies is crucial.

Another limitation of the approach is that it can only be applied to the sample-related ROI pixels. Thus, when selecting these pixels, the spatial information necessary to identify possible non-sample-related signals is lost. However, this difficulty can be easily overcome by applying spatial-aware filters, such as SPUTNIK [36], before the recalibration. In this way, most of the signal correlated with regions outside of the ROI is removed before performing the pixels selection. This aspect is crucial for the correct biological interpretation of the observed molecular spatial patterns.

In the future, we will work to extend the method to other types of ion analyzers and will study the possible integration with the usage of external lock mass reference ions.

## Supplementary Information

**Additional file 1.** Details about the MSI pre-processing and mass recalibration algorithm. Results of the comparison between original and recalibrated mass accuracies.

## Data Availability

The Python code is freely distributed under the MIT license and can be downloaded from https://github.com/paoloinglese/DESI_MSI_recal, together with the reference masses databases. Additional results consisting of all annotations from METASPACE, annotated reference masses, number of reference masses per pixel and model coefficients are available on Open Science Framework at https://osf.io/q4h76/.

## Footnotes

1 https://github.com/alexandrovteam/pyimzML.

2 https://metaspace2020.eu/.

3 To simplify the notation, we denote the predicted observed masses with the symbol ${\widehat{\mathbf{M}}}_{k}$ instead of ${{\widehat{\mathbf{M}}}^{\#}}_{k}$.

4 For simplicity, we use the index $p$ instead of ${p}_{i}$ to denote the pixel dependency.

5 https://github.com/tommyod/KDEpy.

6 https://github.com/LaRoccaRaphael/MSI_recalibration/blob/master/recalibration/internal_calibrants_generation.ipynb.
